# Life Course Trajectories of Labour Market Participation among Young Adults Who Experienced Severe Alcohol-Related Health Outcomes: A Retrospective Cohort Study

**DOI:** 10.1371/journal.pone.0126215

**Published:** 2015-05-04

**Authors:** Tapio Paljärvi, Pekka Martikainen, Tiina Pensola, Taina Leinonen, Kimmo Herttua, Pia Mäkelä

**Affiliations:** 1 Alcohol and Drugs Unit, National Institute for Health and Welfare, Helsinki, Finland; 2 Population Research Unit, University of Helsinki, Helsinki, Finland; 3 Max Planck Institute for Demographic Research, Rostock, Germany; 4 Health and Work Ability, Finnish Institute of Occupational Health, Helsinki, Finland; 5 Centre of Maritime Health and Society, University of Southern Denmark, Esbjerg, Denmark; The George Institute for Global Health, INDIA

## Abstract

**Background:**

Long-term employment trajectories of young problem drinkers are poorly understood.

**Methods:**

We constructed retrospective labour market participation histories at ages 18–34 of 64 342 persons born in 1969–1982. Beginning from the year of each subject’s 18^th^ birthday, we extracted information from the records of Statistics Finland on educational attainment, main type of economic activity, months in employment, and months in unemployment for a minimum of seven years (range 7–16 years). We used information on the timing of alcohol-related hospitalizations and deaths in the same period to define problem drinkers with early onset limited course, early onset persistent course, and late onset problem drinking.

**Results:**

Early onset limited course problem drinkers improved their employment considerably by age, whereas early onset persistent problem drinkers experienced a constant decline in their employment by age. From the age of 18 to 34, early onset persistent problem drinkers were in employment merely 12% of the time, in comparison with 39% among the early onset limited course problem drinkers, and 58% among the general population.

**Conclusions:**

These results indicate that young adults who were retrospectively defined as having early onset persistent course problem drinking were extensively marginalized from the labour market early on during their life course, and that their employment trajectory was significantly worse compared to other problem drinkers.

## Introduction

Problem drinking [1–4] has the potential to determine life trajectories e.g. by adversely affecting the success in transitions between social roles and statuses [5, 6], such as gaining employment after leaving school [7]. Problem drinking in adolescence and early adulthood [8–10] has been found to be associated with low educational [11–15] and occupational attainment [16, 17], and unemployment [18, 19]. It has, however, remained unclear how the long-term trajectories of labour market participation of young adults who develop a severe and persistent form of problem drinking differ from the trajectories of those who present a more normative pattern in which problem drinking is likely to resolve or remit at adulthood [20, 21]. It would be particularly important to establish whether young adults who develop a severe form of problem drinking relatively early in their life course are able to participate in the labour market after completing their compulsory education, or if they are marginalized from the labour market early on.

Many of those with an early onset problem drinking experience their highest level of symptoms of problem drinking in their early twenties [20], and the majority are likely to recover by adulthood [21]. However, particularly those who progress rapidly to problem drinking after drinking initiation and who present persistent problem drinking over their life course, [8–10] typically have a history of numerous co-occurring and interacting personal and environmental adversities which markedly hinder their capability to function on the labour market [22]. From this point of view, it is probably more fruitful to see problem drinking as a manifestation of proneness to a certain type of behavioural response involving harmful alcohol use [5], rather than seeing problem drinking as a risk factor operating independently. This perspective does not diminish the role of problem drinking as a modifiable and preventable risk factor for adverse employment trajectories and labour market marginalization but it calls for an approach which takes into account the wider context from which problem drinking arises and the factors through which it is maintained [23]. For example, given that many young people experience unemployment before gaining their first employment after school [24], it is possible that at least part of the employment disadvantage of the problem drinkers is mediated by an increased vulnerability to the harmful effects of even short periods of unemployment [25, 26]. Among vulnerable persons the experience of unemployment can potentially act either as a trigger or catalyst for problem drinking [27], which may then lead to cascading health problems and further difficulties in gaining employment [28]. Therefore, understanding of the complex interplay between problem drinking and labour market marginalization among young adults is important in developing targeted interventions aimed to reduce the public health burden related to socioeconomic disadvantage and in reducing health inequalities across population groups [29].

### Aim and design

Because severe forms of problem drinking among young adults are relatively rare in the general population, prospective population-based cohort studies are often not feasible for studying long-term trajectories of labour market participation among these persons. The purpose of this register-based retrospective cohort study is therefore to establish how young adults aged 18–34 years, who were hospitalized or died due to alcohol-related causes, participated in the labour market after they had completed their compulsory basic education. We used the alcohol-related diagnoses recorded in the Hospital Discharge Register and the Cause of Death Register as proxy measures for a severe form of problem drinking. This operationalization is in accordance with the definition of a problem drinker provided by the World Health Organization [30]. In line with the developmental perspective on problem drinking [21, 23, 31], we use the timing of alcohol-related hospitalizations and deaths to determine the trajectories of labour market participation separately for those who experienced an early onset limited course, an early onset persistent course, and a late onset problem drinking. The cohort design enables us to compare the trajectories of labour market participation across these problem drinking groups and the general population when they navigated through comparable age-related transitions in their life course regarding educational attainment and employment. Information on labour market participation was collected each year beginning from each subject’s 18^th^ birthday for a minimum of seven years and up to 16 years. Comparison with the general population will give us an idea of the differences in relation to the normative trajectory of labour market participation. Using this novel design, we do not aim to establish the causal role of problem drinking in determining labour market participation but we aim to provide an insight to the typical group-level employment trajectories of persons among whom alcohol plays a substantial negative role in their life course. This information could be used in developing more effective interventions aimed to promote employability among individuals who are particularly vulnerable to the severe and persistent negative life course effects of problem drinking.

## Data and Methods

### Ethics statement

The sampling and data linkage was approved by the ethics committee of Statistics Finland.

### Study population

The original register-based study data consists of an 11% random sample of all persons who were living in Finland during the years 1987–2007. For the purpose of this study we selected those who were aged 18 at entry, and followed them up for a minimum of seven years (beginning from the age of 18 and up to the age of 34, range 7–16 years). An additional sample of all deaths that occurred during the study period was drawn so that 80% of the persons who died during the study period were identified. This was the maximum representation of deaths retrievable from the records of Statistics Finland due to data protection rules. Information from the nation-wide registers of Statistics Finland (causes of death, sociodemographic factors, and indicators of labour market participation) and the National Institute for Health and Welfare (causes of hospital admissions) were linked to the data. Statistics Finland carried out the sampling and data linkage using a unique personal identity code issued to all Finnish residents and available in all registers used in this study. All data were anonymized and de-identified by Statistics Finland prior to analyses.

We constructed retrospective cohorts of persons born in 1969–1982 who remained in Finland after the age of 17 (N = 76 609). Persons who immigrated or emigrated during the study period were excluded from the data (n = 7721). To ensure that all participants had the opportunity to experience comparable age-related transitions in their life course regarding educational and occupational attainment, persons who died before the age of 25 were also excluded (n = 4546). All deaths, therefore, occurred in 1994–2007 at ages 25–34. Hospitalizations were not used in determining eligibility and were thus allowed to occur at any time up to the age of 34. The final study data consisted of 64 342 persons.

### Retrospective proxy measures of problem drinking

We used the alcohol-related diagnoses recorded in the Hospital Discharge Register and the Cause of Death Register as surrogate/proxy measures for problem drinking. Alcohol-related hospitalizations and deaths were identified using the International Classification of Diseases, Finnish modification, ninth (ICD-9) and tenth revisions (ICD–10) codes. For ICD-9 we used the following codes: 291, 303, 3050, mental and behavioural disorders due to use of alcohol; 3575, alcoholic polyneuropathy; 4255, alcoholic cardiomyopathy; 5353, alcoholic gastritis; 5710–5713, alcoholic liver disease; 5770D–5770F, 5771C–5771D, alcoholic pancreatitis; 980, toxic effect of alcohol; E851, accidental poisoning by alcohol. For ICD-10 we used the following codes: F10, mental and behavioural disorders due to use of alcohol; G312, degeneration of nervous system due to alcohol; G4051, epileptic seizures related to alcohol; G621, alcoholic polyneuropathy; G721, alcoholic myopathy; I426, alcoholic cardiomyopathy; E244, alcohol-induced pseudo-Cushing syndrome; K292, alcoholic gastritis; K70, alcoholic liver disease; K852, alcohol-induced acute pancreatitis; T51, toxic effects of alcohol; X45, accidental poisoning by and exposure to alcohol. We used primary and secondary diagnoses in identifying alcohol-related hospitalizations, and underlying and contributory causes of death in identifying alcohol-related deaths in order to improve the coverage in identifying problem drinkers. By using secondary diagnoses of hospitalizations and contributory causes of deaths, we identified e.g. persons who had alcohol intoxication or alcohol dependence recorded as a contributing factor. A total of 2600 persons were retrospectively defined as problem drinkers based on the above criteria.

#### Time-ordered patterns of problem drinking

Alcohol-related hospitalizations occurring before the age of 25 were considered as indicators of early onset problem drinking [31]. Early onset limited course problem drinking was defined as having been hospitalized due to alcohol-related causes before the age of 25 but not having been re-hospitalized or died due to alcohol-related causes at ages 25–34 years (n = 457). In defining this pattern, we also took into account competing causes of death so that those who died, e.g. from injuries, without alcohol involvement between ages 25 and 34 were excluded from this group. Early onset persistent course of problem drinking was defined as having been hospitalized due to alcohol-related causes before the age of 25 and having been re-hospitalized or died due to alcohol-related causes between ages 25 and 34 (n = 526). In addition, those excluded from the early onset limited course group were included in this group (i.e. those with alcohol-related hospitalization before the age of 25 and death without alcohol involvement between ages 25 and 34). Those who had their first alcohol-related hospitalization or who died due to alcohol-related causes between ages 25 and 34 were defined as having a late onset problem drinking (n = 1617). Table 1 shows the combinations of alcohol-related hospitalizations and deaths at different age periods used in defining the time-ordered patterns of problem drinking.

**Table 1:** Time-ordered patterns of alcohol-related hospitalizations and deaths used in defining the developmental subtypes of severe form of problem drinking.

	Alcohol-related hospitalization before the age of 25	Alcohol-related hospitalization between ages 25 and 34	Alcohol-related death between ages 25 and 34	Death without alcohol involvement between ages 25 and 34	Number of subjects (n = 2600)
Early onset limited	1	0	0	0	457
Early onset persistent	1	1	0	0	133
	1	0	1	0	100
	1	1	1	0	122
	1	1	0	1	77
	1	0	0	1	94
Late onset	0	1	0	0	569
	0	0	1	0	696
	0	1	1	0	202
	0	1	0	1	150

Number 1 indicates inclusion.

### Analysis variables

Beginning from the year of each subject’s 18^th^ birthday, we extracted information from the records of Statistics Finland on indicators relevant to labour market participation; educational attainment, main type of economic activity, months in employment, and months in unemployment. The highest level of education attained after the compulsory basic education was recorded according to the International Standard Classification of Education (ISCED) coding. Among those who were not educated beyond the compulsory basic education, the ISCED coding was lower secondary education (level 2). In Finland, this typically corresponds to 9–10 years of schooling. Information on main type of economic activity indicated whether the person was employed, unemployed, student, pensioner, conscript, or other outside the labour force at the end of a given year. This is later referred to as labour market participation. Persons not in employment, education or training, “NEETs” were defined as those whose main activity at the end of a given year was not one of: employed, student, or conscript. Time outside the labour force was defined as months not being employed or unemployed. We used information on living arrangement and household composition to determine whether the person lived alone or not. Leaving parental home and living alone at an early age, particularly if not because of attending school, is potentially an indicator of various negative life course characteristics, e.g. in the childhood family environment [32, 33], and can therefore reflect early risk factors for negative labour market outcomes.

### Statistical analysis

The main results are based on comparisons across the three groups reflecting retrospectively defined time-ordered patterns of problem drinking, in particular in terms of contrasting early onset limited course with early onset persistent course of problem drinking. Comparisons against the general population provide a quantification of the overall disadvantage in relation to the normative life course trajectories.

We used generalized estimating equations (GEE) in analyzing the average time in employment, unemployment, and time outside labour force, while adjusting for the effects of sex, cohort, and age at death/end of follow-up. By using GEE we estimated the group-level employment trajectories. This method adjusts the standard errors for the within-subject clustering of data over repeated measurements (7–16 measurement points). We used an autoregressive correlation structure on the assumption that within subject measurements closer in time are more highly correlated than measurements further apart. The results from these models are presented as estimated marginal means with their 95% confidence intervals. Analysis weight variable was used in all the analyses to correct for the different sampling probability between survivors (11%) and the deceased (80%). SAS/STAT software's GENMOD procedure was used to perform the GEE analyses.

## Results

### Profile of alcohol-related hospitalizations and deaths

Men were overrepresented among those who were hospitalized or died due to alcohol-related causes (80%). Among men and women, the first alcohol-related hospitalization before the age of 25 occurred at a mean age of 19.6 years (range 10–24 years, SD = 3.7), the mean age for the first alcohol-related hospitalization between ages 25 and 34 was 28.5 years (SD = 3.0), and the mean age for alcohol-related death was 28.9 years (range 25–34 years, SD = 1.4). The majority (77%) of alcohol-related hospitalizations were related to diagnoses of mental and behavioural disorders, and the second largest diagnostic group was injuries/external causes (14%). The relative contribution of alcohol-related injuries/external causes was largest among early onset limited course problem drinkers (30%) and lowest among late onset problem drinkers (6%). Of the alcohol-related deaths, 82% were related either to suicide (40%), poisoning (14%), or injuries/other external causes (28%).

### Life course characteristics

Table 2 shows the life course characteristics relevant to labour market participation. Compared to the general population, persons who were retrospectively defined as problem drinkers had a markedly lower educational attainment. About half (49% of the men and women) of these problem drinkers, and as much as 64% of early onset persistent course problem drinkers, did not acquire formal education beyond the compulsory basic education, whereas only about one in ten (12% of the men and women) in the general population had equally low educational attainment.

**Table 2:** Life course characteristics of the study population.

			Patterns of problem drinking^a^		
	General population (n = 61 742)	All patterns of problem drinking combined (n = 2600)	Early onset limited course (n = 457)	Early onset persistent course (n = 526)	Late onset (n = 1617)
Men	31892	2084	306	433	1345
Basic education only (%)	14 (14–15)	51 (50–52)	46 (45–48)	66 (64–68)	50 (49–51)
At the age of 18 (%)					
Living alone	4 (3–4)	11 (10–13)	11 (7–14)	19 (13–24)	10 (8–12)
Labour market participation^b^					
In education	67 (66–67)	42 (39–45)	46 (40–52)	33 (26–39)	43 (39–46)
Employed	22 (22–23)	25 (23–28)	23 (18–28)	24 (18–30)	27 (24–30)
Unemployed	8 (8–9)	25 (22–27)	23 (18–28)	30 (24–37)	24 (21–28)
Other non-employed	3 (2–3)	8 (6–9)	8 (5–11)	13 (9–18)	6 (4–8)
NEET^c^	11 (11–12)	33 (30–35)	31 (26–36)	43 (37–51)	30 (27–34)
At the age of 24 (%)					
Living alone	29 (29–30)	42 (39–45)	41 (35–46)	51 (44–57)	40 (36–44)
Labour market participation^b^					
In education	22 (22–23)	13 (11–15)	13 (10–17)	10 (6–14)	14 (11–16)
Employed	61 (60–61)	31 (29–34)	34 (28–39)	14 (9–19)	34 (31–38)
Unemployed	13 (13–14)	37 (34–39)	29 (24–34)	44 (37–51)	39 (35–42)
Other non-employed	4 (3–4)	19 (17–21)	24 (19–29)	32 (26–39)	13 (10–15)
NEET^c^	17 (17–18)	56 (53–58)	53 (47–58)	76 (70–82)	52 (48–55)
Women	29850	516	151	93	272
Basic education only (%)	10 (9–10)	47 (46–49)	43 (41–46)	62 (58–66)	47 (44–49)
At the age of 18 (%)					
Living alone	6 (5–6)	20 (16–24)	23 (16–30)	29 (16–42)	15 (10–20)
Labour market participation^b^					
In education	70 (69–70)	46 (41–51)	48 (40–56)	38 (24–52)	45 (38–52)
Employed	22 (21–22)	26 (22–31)	26 (19–34)	28 (15–41)	26 (19–32)
Unemployed	5 (4–5)	19 (15–23)	19 (12–25)	18 (7–29)	20 (14–26)
Other non-employed	3 (2–3)	9 (6–12)	7 (3–11)	15 (5–26)	9 (5–14)
NEET^c^	8 (8–9)	28 (23–33)	26 (18–32)	33 (20–47)	29 (22–36)
At the age of 24 (%)					
Living alone	27 (26–27)	34 (30–39)	28 (21–36)	42 (28–56)	38 (31–45)
Labour market participation^b^					
In education	22 (22–23)	17 (13–21)	19 (13–25)	24 (12–37)	14 (9–19)
Employed	55 (54–55)	34 (30–39)	42 (34–50)	21 (9–32)	30 (24–38)
Unemployed	12 (12–13)	23 (19–27)	13 (7–17)	29 (16–41)	32 (25–38)
Other non-employed	11 (10–11)	25 (21–30)	26 (19–34)	26 (14–38)	24 (17–30)
NEET^c^	23 (23–24)	48 (43–53)	39 (31–47)	55 (48–63)	55 (41–69)

Finnish men and women aged 18–34 years. 95% confidence interval (CI) in the parenthesis.

^a^Defined retrospectively based on the timing of alcohol-related hospitalizations and/or deaths.

^b^Status at the end of year.

^c^Those not in employment or education, i.e. unemployed and other non-employed combined.

The proportion of persons living alone at the age of 18 was around three-fold higher among retrospectively defined problem drinkers, compared to the general population. Women were more frequently living alone than men. Among retrospectively defined problem drinkers, the proportion of persons living alone at the age of 18 was lowest among those who were defined as having late onset problem drinking, and highest among those defined as having early onset persistent course problem drinking.

At the age of 18, around one in three (31%) of the men and women who were retrospectively defined as problem drinkers were not in employment or in education (NEETs), in comparison with around one in ten among the general population (10%). By the age of 24, the proportion of NEET men and women among problem drinkers had increased to around one in two (52%), in comparison with one in five among the general population (20%). In all three problem drinking groups, the rate of moving outside the labour force was higher than the rate of moving to unemployment. For example, of the male early onset persistent course problem drinkers, 30% were unemployed at the age of 18, and this proportion increased to 44% by the age of 24 (1.5-fold increase), whereas the proportion outside the labour force increased in the same period from 13% to 32% (2.5-fold increase).

The results suggest that the transition from formal education to employment was markedly less successful among problem drinkers compared to the general population. Even if the proportion on persons in education decreased between ages 18 and 24 in a comparable fashion in the various groups, the parallel increase in the proportion of persons in employment was markedly lower among those who were retrospectively defined as problem drinkers (1.3-fold increase from 26% to 33%) than among the general population (2.6-fold increase from 22% to 58%).

### Accumulated employment

**Fig 1:**
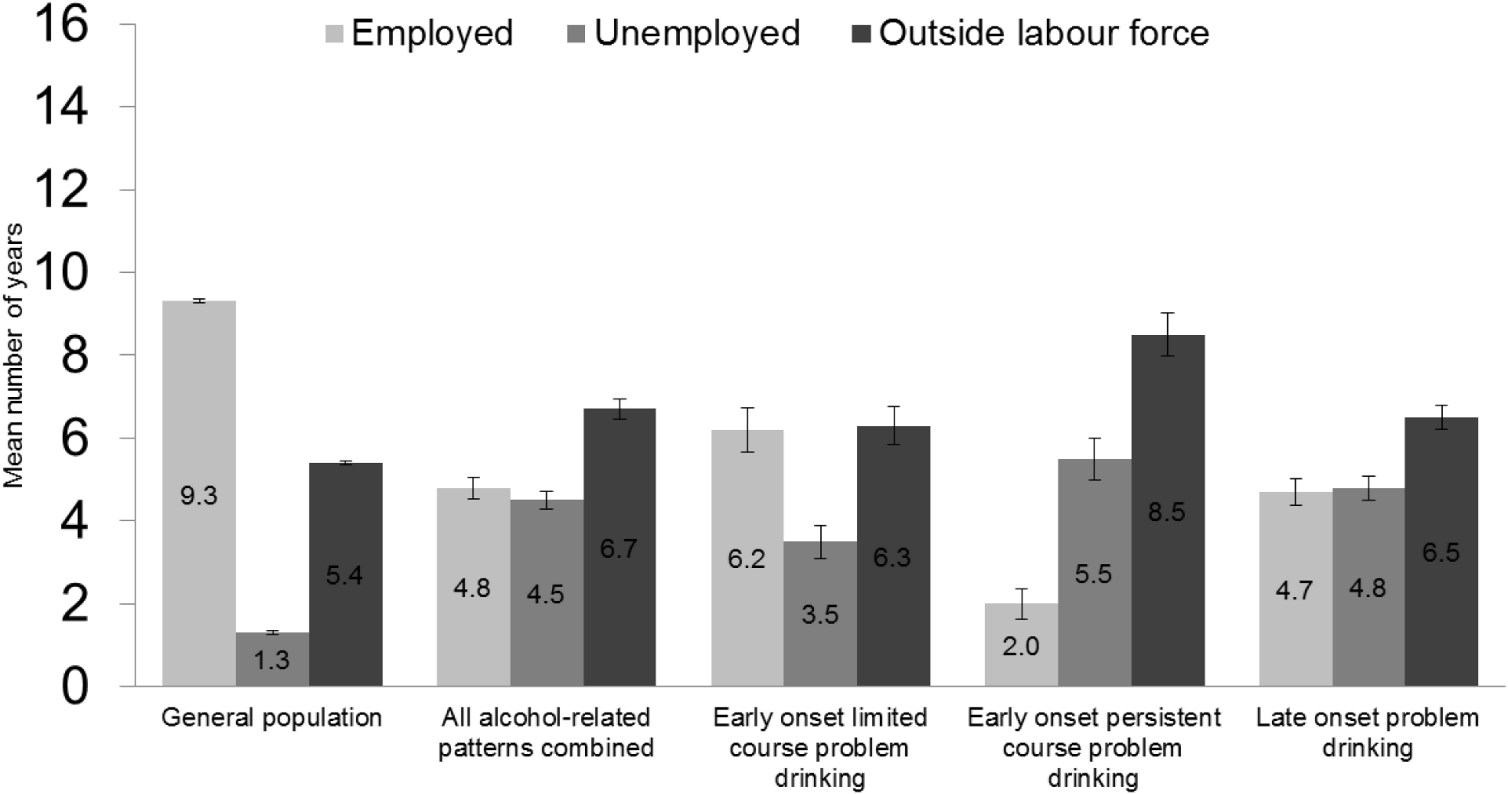
Average number of years employed, unemployed, and outside the labour force over the 16-year follow-up period by retrospectively defined patterns of problem drinking. Means adjusted for cohort, sex, and age at the time of death/end of follow-up. Error bars represent the 95% confidence intervals. Finnish men and women aged 18–34 years.

Fig 1 shows the average number of years employed, unemployed, and outside the labour force over the 16-year study period from the age of 18 to 34, adjusted for cohort, sex, and age at the time of death/end of follow-up. Persons who were retrospectively defined as problem drinkers were on average 4.5 years less in employment over the 16-year period from the age of 18 to 34 years than the general population (4.8 years, 95%CI = 4.6–5.1 vs. 9.3 years, 95%CI = 9.3–9.4). However, there were marked differences in the accumulated level of employment within the retrospectively defined problem drinkers. The early onset persistent course problem drinkers were in employment on average 2.0 years (95%CI = 1.6–2.4) over the 16-year study period, which is one-third of the accumulated employment among the early onset limited course problem drinkers (6.2 years, 95%CI = 5.8–6.8), and also clearly less than that among the late onset problem drinkers (4.7 years, 95%CI = 4.5–5.2).

All in all, the early onset persistent course problem drinkers were employed 12% (2.0/16), unemployed 34% (5.5/16), and outside the labour force 53% (8.5/16) of the time between ages 18 and 34. The early onset limited course problem drinkers were employed 39%, unemployed 22%, and outside the labour force 39% of the time between ages 18 and 34. The respective proportions among the general population were 58%, 8%, and 34%. It should be noted here that being outside the labour force among the general population was mainly related to being in formal education, whereas among those who were retrospectively defined as problem drinkers, being in education contributed much less to the time outside the labour force, as shown in Table 2.

### Employment trajectories

**Fig 2:**
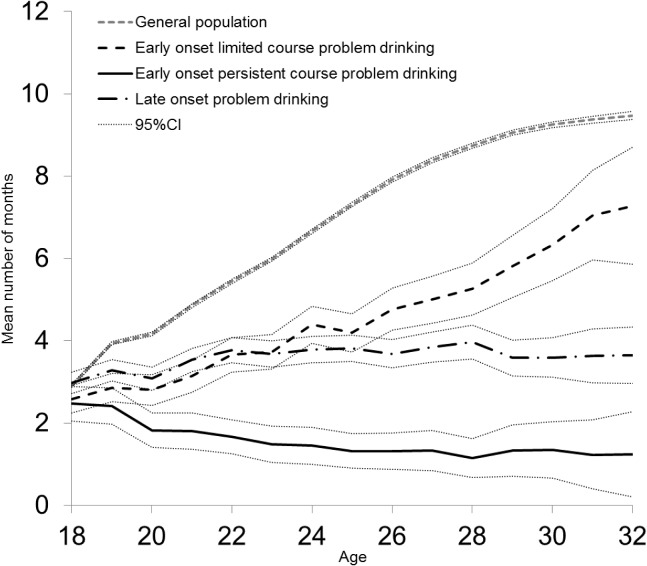
Average number of months of employment at each year of age by retrospectively defined patterns of problem drinking. Means adjusted for cohort, sex, and the age at the time of death/end of follow-up. Finnish men and women.

Fig 2 shows the group-level employment trajectories expressed as the mean annual number of months of employment from the age of 18 to 32, adjusted for cohort, sex, and age at the time of death/end of follow-up. At the age of 18, there was no difference in the level of employment across any of the groups. However, all three retrospectively defined problem drinking groups showed a distinctive long-term employment trajectory significantly different from that of the general population.

The employment trajectory of the early onset limited course problem drinkers, and the late onset problem drinkers followed a comparable trajectory up to the age of 25, after which the trajectories diverged statistically significantly. By the age of 26, the increasing employment trajectory of the late onset problem drinkers had levelled off, whereas the employment trajectory of the early onset limited course problem drinkers continued to increase. By the age of 32, the difference in the average level of employment between these groups had increased to two-fold (7.3, 95%CI = 5.9–8.7 vs 3.7, 95%CI = 2.9–4.3).

In contrast with the other two retrospectively defined problem drinking groups, the early onset persistent course problem drinkers showed a declining employment trajectory. Their average level of employment was one-third at the age of 24 and at the age of 32 one-sixth, compared to the level of employment of the early onset limited course problem drinkers.

## Discussion

### Summary of main findings

We established how young adults aged 18–34 who were retrospectively defined as problem drinkers based on the alcohol-related harm they experienced, participated in the labour market after completing their compulsory basic education. All models were adjusted for the effects of cohort, sex, and age at the time of death/end of follow-up. About half of these young problem drinkers were disengaged from formal education at the age of 18. As a result, the proportion of persons with compulsory basic education as the highest educational attainment was four-fold higher among them compared to the general population. Furthermore, compared to the general population, their transition from formal education to employment was significantly less successful, which was evidenced e.g. by a disproportionately high level of unemployment already at the age of 18, and the markedly lower rate of gaining employment after the age of 18. During the age period 18–34, the early onset persistent course problem drinkers were in employment merely 12% (i.e. two years of the 16-year follow-up period) of the time, in comparison with 39% of the time in employment among the early onset limited course problem drinkers, and 58% among the general population.

Despite being disadvantaged in relation to the general population, those who belonged to the early onset limited course problem drinking group improved their employment considerably by age, whereas those in the early onset persistent problem drinking group experienced a constant decline in their employment. In particular, young persons who were retrospectively defined as persistent problem drinkers were, therefore, markedly disadvantaged in the labour market in terms of their ability to gain employment, compared to other problem drinkers and the general population.

### Methodological considerations

Severe forms of problem drinking are relatively rare among young adults in the general population. For example in 2007, of all the alcohol-related hospitalization in the general population 3.9% were among persons under 25 and 9.7% among persons under 35 years [34]. Our register-based retrospective study design enabled us to capture a relatively large number of young persons with alcohol-related hospitalizations and deaths, and who could thus be retrospectively defined as problem drinkers [30]. This increased precision of estimation in our results and increased statistical power to detect differences. Self-report bias and potential non-response bias did not affect our results because all information was collected from administrative registers. The cohort design enabled us to compare the trajectories of labour market participation across the retrospectively defined problem drinkers and the general population, during an age period when all experienced comparable age-related transitions in their life course regarding educational attainment and employment.

However, because the definition of problem drinking was based on alcohol-related hospitalizations and deaths, the results apply only to those problem drinkers who are at risk of experiencing similar alcohol-related health outcomes. On the continuum of harm, hospitalizations and deaths represent the most severe end of the continuum, and therefore those who are at risk of experiencing less severe alcohol-related harm, such as outcomes leading only to ambulatory care, could have different trajectories of labour market participation. A limitation is that we did not have information on actual alcohol consumption, which could have been used in establishing the timing and level of harmful alcohol use more accurately. Our time-ordered patterns of problem drinking, however, enabled us to control at least some of the issues related to timing between problem drinking and trajectories of labour market participation. For example, the first alcohol-related hospitalization in our data occurred at a mean age of 19.6 years for those who were defined as having an early onset problem drinking, which means that the first alcohol-related hospitalization occurred on average at the beginning of the follow-up period for labour market participation.

We excluded those who died before the age of 25 because persons who died at very young age had very limited opportunity to gain high educational attainment and participate in the labour market. The non-employment estimates for the retrospectively defined problem drinkers are therefore likely underestimates rather than overestimates. We controlled cohort effects statistically in the GEE models by adjusting for birth year but the potential effects of residual confounding should still be kept in mind when interpreting the results because birth year is only a proxy for e.g. the differences in the societal context affecting the success of different birth cohorts entering labour market.

### Interpretation of main results

Our finding of the low educational level and high level of unemployment among retrospectively defined problem drinkers is in line with findings in other populations using different criteria for problem drinking [11–15, 19]. Our results thus corroborate and extend understanding on two important aspects of the typical life course of young problem drinkers who are at risk of experiencing severe alcohol-related health outcomes. First, the low educational attainment meant that these persons had limited opportunities in terms of gaining high occupational attainment. Second, their early disengagement from formal education did not predominantly lead to an early entry to employment [35].These two interrelated factors, i.e. low educational attainment and youth unemployment, are major determinants of labour market disadvantage later in life [19, 36], and can thus potentially explain a considerable part of the employment disadvantage accumulated over the life course of problem drinkers [37]. This employment disadvantage is intensified in contexts, such as Finland, where the majority of the population in the cohort studied has high educational attainment and where there are limited employment opportunities available for unskilled workers. Furthermore, increased labour market demands in terms of workforce performance means that persons whose work ability is reduced due to problem drinking are likely facing even more difficulties in the labour market in the future than what was the case previously.

The worst employment trajectory was seen among the early onset persistent course problem drinkers. In this group, the role of problem drinking is more strongly indicated than among early onset limited course problem drinkers or late onset problem drinkers. It is therefore probable that alcohol played a substantial negative causal role in the life course of these persons. However, particularly those who progress rapidly to problem drinking after drinking initiation and who present persistent problem drinking over their life course [8–10] are frequently characterised by multiple co-occurring adversities, including psychological dysregulation defined as behavioural disinhibition and negative affect [1, 23], and familial adversities, such as parental history of alcohol problems, and various aspects of social and economic disadvantage in the childhood family environment [3, 4]. Life course trajectories among these persons are, therefore, determined not only by problem drinking but a constellation of wider individual and environmental factors interacting with problem drinking. The various intertwined mechanisms underlying labour market disadvantage makes it difficult or even impossible to disentangle the separate effects of the individual factors, such as the independent effect of problem drinking on employment trajectories.

The employment trajectory among the early onset limited course problem drinkers in comparison with the other two problem drinking groups suggested that persons who are able to desist from a severe form of problem drinking are capable of improving their employment considerably over their life course. However, the employment trajectories deviated between the early onset limited course problem drinkers and the late onset problem drinkers only after the age of 25. An important unanswered question, therefore, is why the employment trajectories did not differ for the favour of the late onset group during the age period before the age of 25, in comparison with the early onset limited course problem drinking group. Comparison with the general population shows that the late onset problem drinking group could have had much potential for improvement in their level of employment before the age of 25. One possible explanation for the non-significant difference between these two problem drinking groups is that below the age of 25, the various correlates of problem drinking are relatively more important in determining the success in competing for the limited employment opportunities available for low skilled youth than problem drinking in itself. Another possible explanation is that those defined as having late onset problem drinking were already consuming alcohol in excessive and disruptive manner, but without severe health outcomes and were thus not captured in the early onset group by using our definition of problem drinking based on alcohol-related hospitalizations. Future studies with more detailed measurement of problem drinking and its correlates should aim to shed more light on these differences.

### Implications

An earlier Finnish study found that the majority of older retrospectively defined problem drinkers, i.e. of persons who died due to alcohol-related causes aged 45–64, were in employment up to ten years before death [38]. This indicated that these older retrospectively defined problem drinkers were not extensively marginalized from the labour market earlier in their life course. The present study among younger retrospectively defined problem drinkers, however, indicates a somewhat different conclusion. In terms of the level of employment, there is considerable potential for improvement among these young problem drinkers, but it remains unclear whether this potential could be capitalized on by interventions, because of the uncertainty about their functional capability for work [39, 40].
